# Permeability-Based Measurement of Nanobody Binding to Nuclear Pore Complexes by Transient Scanning Electrochemical Microscopy

**DOI:** 10.1021/acs.analchem.6c04319

**Published:** 2026-09-24

**Authors:** Moghitha Parandhaman, Siao-Han Huang, Kathryn G. Dzurik, W. Seth Childers, Shigeru Amemiya

**Affiliations:** Department of Chemistry, 6614 University of Pittsburgh, 219 Parkman Avenue, Pittsburgh, Pennsylvania 15260, United States

## Abstract

Nucleocytoplasmic macromolecular transport through the nuclear pore complex (NPC) is mediated selectively and efficiently by protein–protein interactions, thereby regulating gene expression and the cell cycle. The NPC transport barrier is formed by biomolecular condensates of nucleoporins (nups) rich in hydrophobic phenylalanine–glycine (FG) repeats. Herein, we develop the transient mode of scanning electrochemical microscopy (SECM) to measure NPC interactions with a nanobody protein targeting the non-FG site of the crucial barrier-forming FG-rich nup, Nup98. The small nanobody, which was recombinantly expressed and purified, diffuses into the NPC and partially blocks the transport of an FG-binding polycationic peptide, protamine. The lowered permeability is measured more sensitively by transient SECM than by the traditional steady-state counterpart under our experimental conditions. A dissociation equilibrium constant between the nanobody and Nup98 in the authentic NPC is determined from the dependence of polypeptide permeability on nanobody concentration. The resulting dissociation constant of 7.7 nM is significantly higher than that of 1.7 nM measured for isolated Nup98 with the nanobody attached to the optical sensor surface in biolayer interferometry. The higher dissociation constant of nanobody–Nup98 interactions in the NPC is consistent with the enthalpically and entropically unfavorable partitioning of the nanobody into the condensate of hydrophobic and disordered nups. The powerful SECM method will be applicable to investigate a variety of NPC-binding proteins with the authentic NPC, thereby advancing our understanding of protein–NPC interactions and functions.

## Graphical abstract

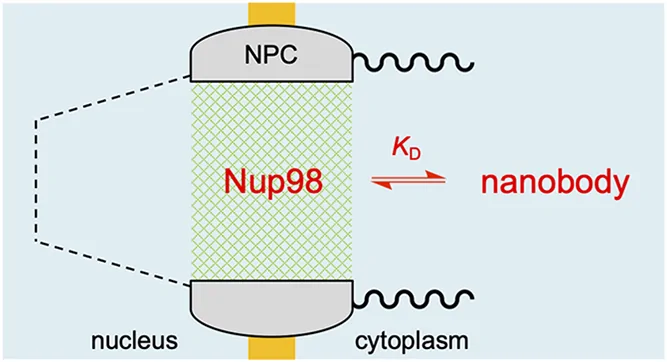

## Introduction

Various analytical methods have been developed and applied to investigate the structural^1^ and functional^2^ roles of protein–protein interactions in macromolecular transport through the nuclear pore complex (NPC). The NPC is a large protein complex (∼120 MDa) that comprises multiple copies of 30 distinct nucleoporins (nups) and perforates the double-membrane nuclear envelope (NE) of a eukaryotic cell.^1^ The NPC solely transports proteins and RNAs between the nucleus and the cytoplasm to regulate gene expression and the cell cycle,^3^ thereby being linked to many human diseases.^4–6^ The nups, rich in hydrophobic repeats of phenylalanine and glycine (FG), form disordered biomolecular condensates within the nanopore and prevent the passive transport of large macromolecules (typically >40 kDa^7^). Large biomacromolecules that are otherwise passively impermeable can be chaperoned as complexes with nuclear transport receptors that interact with FG domains.^8^ Receptor-mediated transport is facilitated by a more hydrophobic cargo, which interacts more favorably with hydrophobic transport barriers.^9^ By contrast, the NPC is blocked by neurotoxic proteins with arginine-containing dipeptide repeats,^10^ which weakly bind to, but slowly dissociate from FG nups, clogging the nanopore.^11,12^

Herein, we advance the transient mode of scanning electrochemical microscopy^13–15^ (SECM) to quantify protein–protein interactions between the NPC and an antinup nanobody. Nanobodies are engineered from camelid-derived antibodies and consist of only two heavy chains, lacking the two light chains typically found in antibodies.^16,17^ The antigen-interacting surface of nanobodies (∼15 kDa) is enlarged to match that of immunoglobulin G (∼150 kDa), thereby recognizing antigens as tightly and selectively as the much larger antibody. Advantageously, nanobodies against FG-rich nups are small enough^18,19^ to diffuse into and recognize the interiors of transport barriers, in contrast to impermeable immunoglobulin G, which binds to the barrier exteriors. Specifically, we employ a nanobody that binds the folded non-FG domain of Nup98 as a major barrier-forming FG-rich nup.^20^ The specific interactions of the anti-Nup98 nanobody with the isolated Nup98 have been determined by X-ray crystallography (Figure 1).^21^ Biolayer interferometry was also employed to measure interactions between the surface-conjugated anti-Nup98 nanobody and the Nup98 protein isolated in solution.^20^ Moreover, a fluorescence conjugate of the anti-Nup98 nanobody was located at the periphery of the nanopore using super-resolution fluorescence microscopy.^21^

**Figure 1:**
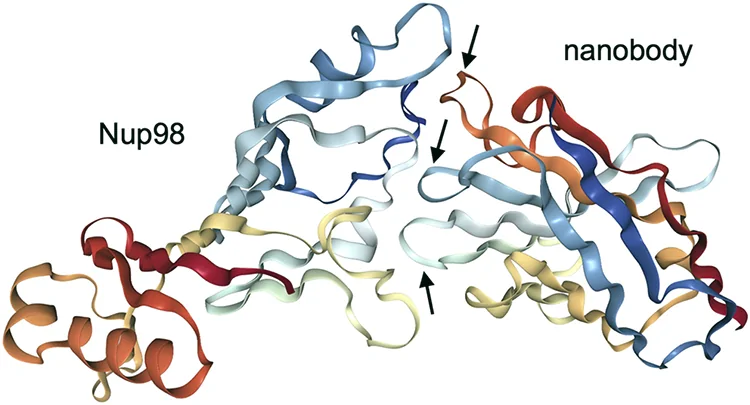
Crystal structure of Nup98 anchor domain in complex with an anti-Nup98 nanobody through antigen-binding loops indicated by arrows. This image was adapted from an image from the RCSB PDB of PDB ID pdb_00005e0q.^21^

Experimentally, we employ transient SECM to measure NPC-mediated transport of polycationic peptide protamine, used as a probe, in the presence of the anti-Nup98 nanobody, which serves as a transport inhibitor. Recently, we demonstrated that arginine-rich protamine mimics neurotoxic proteins with arginine-containing dipeptide repeats, thereby interacting with the FG units of barrier-forming nups.^11^ In the transient mode, protamine was pre-equilibrated with the NPC and then dissociated from the nanopore and monitored both chronoamperometrically using a protamine-selective micropipet tip. The resulting current response of the SECM tip was analyzed^22^ to fully and quantitatively characterize NPC–protamine interactions. Specifically, a dissociation rate constant, *k*_off_, as a measure of the NPC permeability to protamine, an equilibrium association constant, β, and the concentration of protamine-binding sites, Γ_s_, were determined precisely. In this work, the tip-induced dissociative transport of protamine through the NPC is hindered by the nanobody. The dependence of the lowered dissociation rate constant on the higher nanobody concentration yields the dissociation constant, *K*_D_, of Nup98–nanobody interactions in the NPC. Advantageously, the transient SECM method is more sensitive than the traditional steady-state counterpart^23–25^ under our experimental conditions, thereby detecting the partial blockage of the NPC by the small nanobody.

Technologically, this work reports the first application of SECM to quantitatively measure protein–protein interactions. SECM has been applied extensively to detect protein–protein interactions^26^ for electrochemical immunoassay.^27^ Moreover, SECM has detected interactions between a DNA-binding protein and a DNA monolayer conjugated to the electrode surface.^28^ Furthermore, we enabled nanoscale SECM imaging of single NPCs plugged with ribonucleoporins.^29^ These steady-state studies, however, did not measure the strength of protein–protein or protein–nucleic acid interactions. By contrast, we employ transient SECM to determine the dissociation constant of a nanobody bound to Nup98 in the NPC. This dissociation constant is significantly higher than that of isolated Nup98 with the nanobody conjugated to the optical sensor surface in biolayer interferometry.^20^ The larger dissociation constant in the NPC reflects the enthalpically and entropically unfavorable partitioning of the nanobody into the condensed barrier of hydrophobic, highly disordered FG-rich nups. Our SECM method is complementary to widely used optical and fluorescence methods, where target proteins must be isolated from condensates,^30^ or conjugated to FG-interacting fluorophores,^9,31^ respectively.

## Experimental Section

### Chemicals and Materials

Protamine sulfate from herring (grade III), poly­(vinylpyrrolidone) (PVP; average molecular weight, 40 kDa), nitrobenzene (≥99%), tetradodecylammonium (TDDA) bromide, and *N*,*N*-dimethyltrimethysilylamine were purchased from Sigma-Aldrich (Milwaukee, WI). Potassium tetrakis­(pentafluorophenyl)­borate (TFAB) was obtained from Boulder Scientific (Mead, CO). Dinonylnaphthalene sulfonic acid (Nacure 1052) was a gift from King Industries (Norwalk, CT). The TDDA salts of dinonylnaphthalenesulfonate^32^ (DNNS) and TFAB^33^ were prepared by metathesis. Silicon nitride (Si_3_N_4_) membranes with a 200 nm-thick square microporous region, 10 μm pore diameter, and 1.8 mm length (NX5200DH10) were obtained from Norcada (Edmonton, Canada). A Milli-Q IQ 7003 water purification system (EMD Millipore, Billerica, MA) was used to obtain UV-treated deionized ultrapure water (18.2 MΩ·cm) with total organic carbon of 5–6 ppb.

Full expression and purification protocols for a nanobody against Nup98 are reported in the literature^20,21^ and detailed in the Supporting Information. Briefly, the xNup98-Nb1t (+1Cys) nanobody with a 14xHis tag was recombinantly expressed in *Escherichia coli* Rosetta (DE3) cells using IPTG induction and purified by nickel affinity chromatography. Following initial capture on Ni-NTA resin, the 14×His tag was cleaved by SUMO protease. The NEDP1 SUMO protease was independently expressed in *E. coli* BL21 (DE3) and purified by FPLC-based Ni-affinity chromatography.^34^ The presence of the nanobody in the resulting solution was confirmed by the Bradford assay. A subtractive Ni-NTA step was used to separate the tagless nanobody from the uncleaved nanobody. SDS-PAGE of the final nanobody (Figure 2) was analyzed by Image Lab 6.1 to yield ∼100% band purity. The stock concentration (0.67 ± 0.01 mg/mL) of xNup98-Nb1t (+1Cys) was determined by absorbance at 280 nm using NanoDrop 2000 (Thermo Fisher Scientific, Waltham, MA) with an extinction coefficient of 2.395 × 10^4^ M^–1^·cm^–1^. The concentration was used to calculate a yield of 0.56 mg nanobody per 1L of culture. The absorbance at 260 nm was also measured to ensure the absence of nucleic acid contamination. Plasmids pTP443 and pDG02583 for the nanobody and the protease, respectively, were obtained from Addgene (Table S1) as gifts from Professor Dirk Görlich (Addgene plasmid # 104129; http://n2t.net/addgene:104129; RRID:Addgene_104129; Addgene plasmid # 216361; http://n2t.net/addgene:216361; RRID:Addgene_216361).

**Figure 2:**
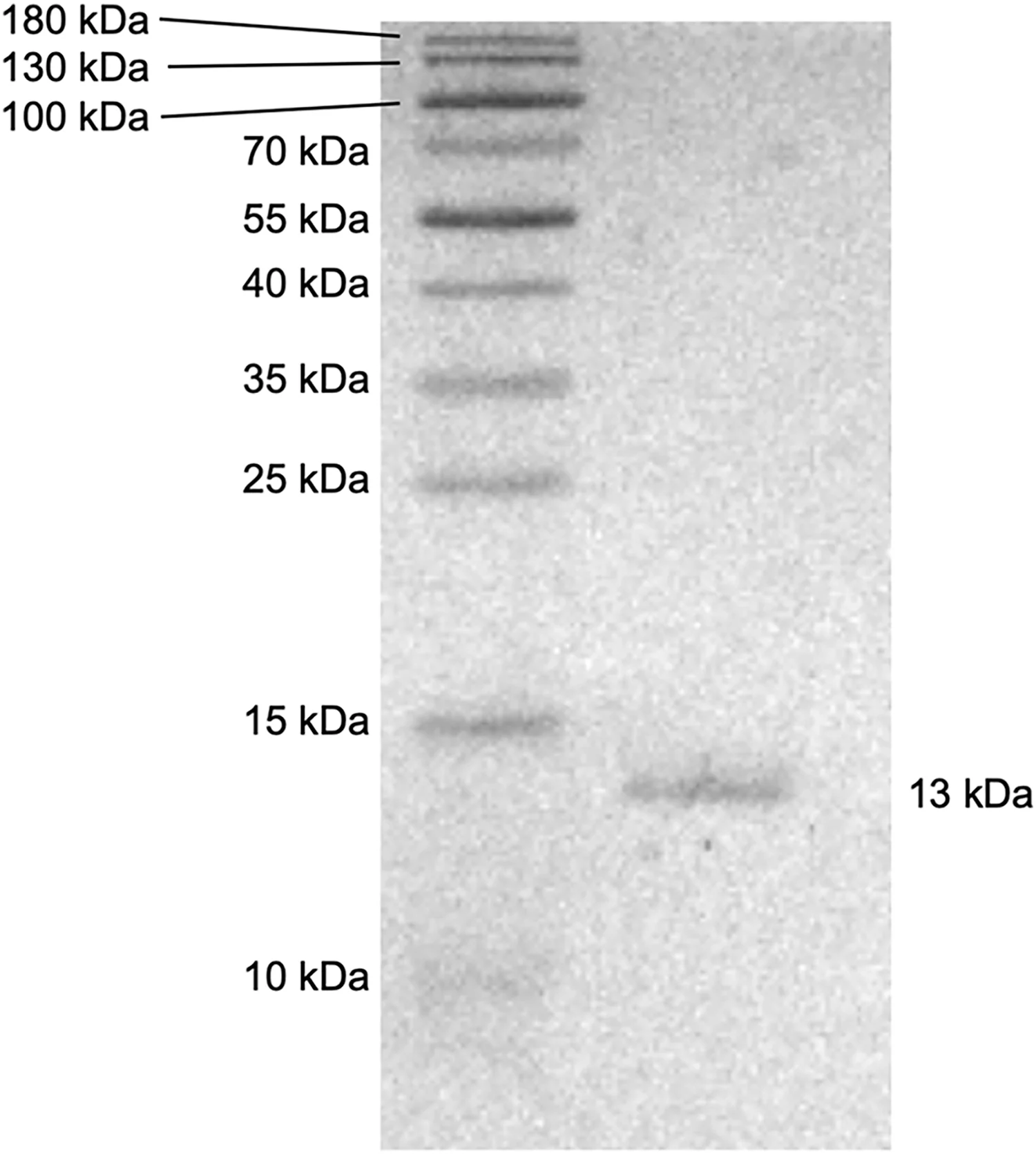
Coomassie-stained SDS-polyacrylamide gel showing the anti-Nup98 nanobody in the purification (right lane) along with molecular weight markers (left lane).

## SECM Measurements

We used SECM to investigate NPC-mediated protamine transport across the NE, spread over a microporous membrane, as reported previously.^11,35^ A home-built SECM instrument^36^ with a potentiostat (CHI 7042E, CH Instruments, Austin, TX) was controlled using a custom LabVIEW program (National Instruments, Austin, TX).^37^ A protamine-selective SECM tip was prepared from a tapered micropipet obtained from borosilicate glass capillaries (o.d./i.d. = 1.0 mm/0.58 mm, 10 cm in length) using a CO_2_-laser capillary puller (model P-2000, Sutter Instrument, Novato, CA) with heat = 470, filament = 4, velocity = 25, delay = 140, and pull = 0. The tapered end of the micropipet was milled by the focused ion beam (FIB) of Ga^+^ (30 keV) using a dual-beam instrument (Scios, FEI, Hillsboro, OR). The tip was milled with a 5 nA Ga^+^ beam and then smoothed with a 100 pA beam. The inner diameter of the tip, 10 μm, was determined by scanning electron microscopy and FIB imaging.^38,39^ FIB-milled glass micropipets were modified with *N*,*N*-dimethyltrimethysilylamine in a vacuum-dried desiccator.^40^ The hydrophobic tips were filled with the nitrobenzene solution of 40 mM TDDADNNS and 100 mM TDDATFAB, and equipped with a Ag electrode (671440, A-M Systems, Sequim, WA). The potential of the Ag electrode was controlled against an aqueous Ag/AgCl reference electrode to drive diffusion-limited protamine transfer across the micropipet-supported nitrobenzene/water interface.

A micropore-supported NE was set up in the SECM cell as reported previously.^11,35,41^ The nucleus was isolated from the oocyte of an adult female *Xenopus laevis* (Ecocyte Bioscience, Austin, TX) to spread the NE on a microporous Si_3_N_4_ membrane. The large nucleus (∼0.4 mm in diameter) was isolated from a stage VI *Xenopus* oocyte in isotonic 1.5% PVP solution of mock intracellular buffer (MIB) at pH 7.4. MIB contains 90 mM KCl, 10 mM NaCl, 2 mM MgCl_2_, 1.1 mM EGTA, 0.15 mM CaCl_2_, and 10 mM HEPES, with free Ca^2+^ buffered at the physiological level of ∼200 nM in oocytes.^42^ The NE was spread in a PVP-free MIB and adhered to the membrane treated with Cell-Tak (Corning, Corning, NY).

A micropipet tip was positioned a short distance above the center of the micropore-supported NE patch in a PVP-free MIB solution containing 10 μM protamine and the nanobody. Tip positioning was guided by SECM approach curves and imaging, with the tip potential set sufficiently negative to drive diffusion-limited protamine transfer.^11,12^ Specifically, a ∼10 μm-diameter micropipet tip approached the nonporous region of the supporting Si_3_N_4_ membrane, thereby hindering protamine diffusion to the tip and lowering the tip current. The micropipet tip was scanned laterally to image the micropore-supported NE patch. The tip current increased as the tip scanned over the NE patch because protamine was transported through NPCs and detected at the micropipet tip. The image was used to position the tip over the center of the NE patch, where the tip-current response was maximal.

Chronoamperograms were measured at short and long distances to eliminate nonfaradaic current, as reported previously.^11,12,43^ Briefly, the chronoamperogram at the long distance was subtracted from that at the short distance. The resulting chronoamperogram matched the simulated one, confirming that the nonfaradaic current was independent of the tip–NE distance and was determined by the resistance between the electrodes inside and outside the micropipet and by the capacitance of the micropipet-supported interface. The capacitance was independent of the tip–NE distance. Moreover, the resistance was dominated by the organic electrolyte solution between the micropipet-supported interface and the Ag electrode in the micropipet, thereby making it independent of the tip–NE distance. The resistance between the interface and the Ag/AgCl electrode in the aqueous solution was negligible.

## Results and Discussion

### Transient SECM of NPC–Nanobody Interactions

We employed the transient mode of SECM to investigate the hindrance of the NPC-mediated protamine transport by the nanobody, thereby measuring NPC–nanobody interactions. In this measurement, a protamine-selective micropipet was positioned near the micropore-supported NE, which was equilibrated with the MIB solution containing 10 μM protamine and 10 nM nanobody (Figure 3A). Both protamine and the nanobody bound to the NPC but at different sites (Figure 3B). The anti-Nup98 nanobody recognizes the C-terminal globular domain of Nup98 (Figure 1B) but not the N-terminal FG domains of Nup98.^21^ By contrast, the guanidinium residue of a protamine molecule engages in cation−π interactions with the aromatic residue of any FG unit with 1:1 stoichiometry.^11,12^ The protamine-selective micropipet was positioned near the NE to amperometrically transfer protamine into the organic ionophore solution in the pipet (Figure 3C). Subsequently, protamine was depleted near the tip and then near the NE and dissociated from the NPCs. The dissociative transport of protamine through the NPC was hindered by the nanobody, thereby lowering the tip current response to protamine. Notably, the dissociation of protamine from the NPC frees FG sites but does not affect the equilibrium binding of the nanobody to the non-FG sites of Nup98. The nanobody was present in the protamine solution during SECM measurements. Because the nanobody–NPC interactions are specific and reversible, as shown later, we did not wash a nanobody-treated nuclear envelope with a nanobody-free solution before SECM experiments.

**Figure 3:**
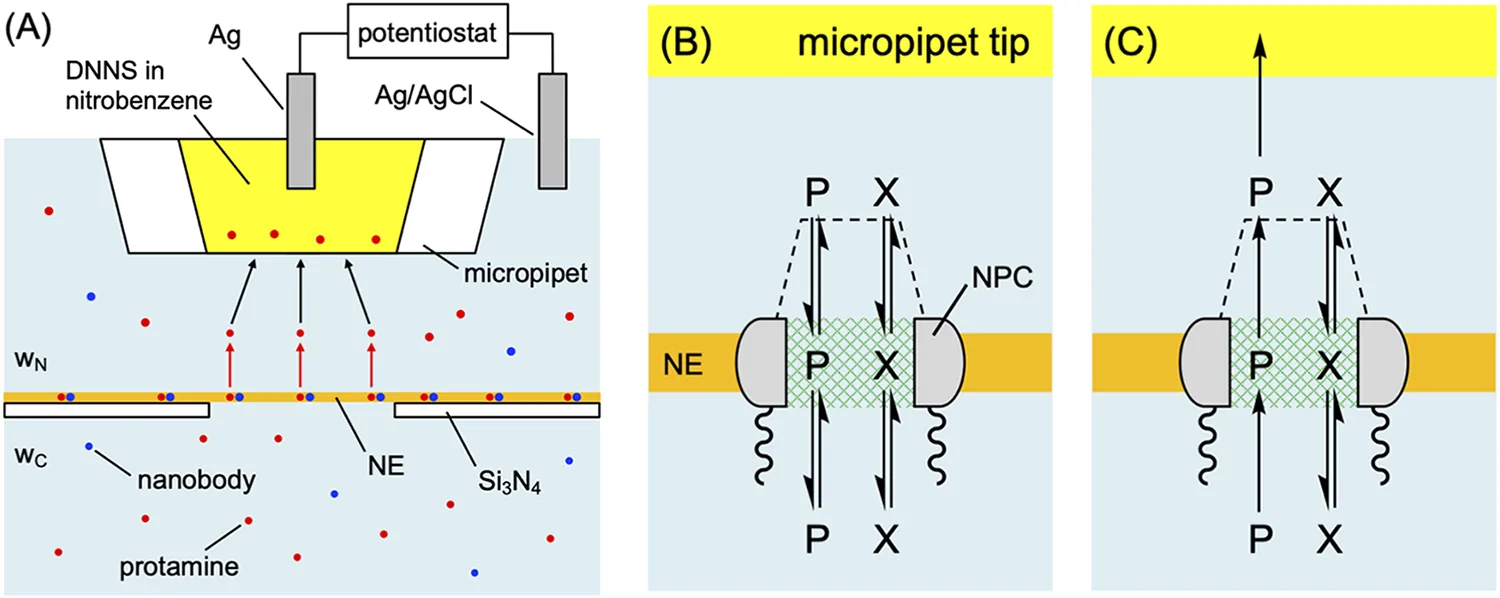
(A) Transient SECM measurement of NPC–protamine interactions in the NE supported by a microporous Si_3_N_4_ membrane. Aqueous solutions on the cytoplasmic and nuclear sides of the NE are indicated by w_C_ and w_N_, respectively. Red and black arrows indicate dissociation and diffusion of protamine, respectively. (B) Pre-equilibrium and (C) induced dissociation of protamine at the NPC with protamine, P, and the nanobody, X.

Experimentally, we investigated NPC interactions with protamine in the presence of the nanobody by comparing the chronoamperometric response of the SECM tip positioned near and far from the NE. The micropipets were filled with a nitrobenzene solution containing DNNS as a negatively charged ionophore for polycationic protamine.^44,45^ Initially, the tip potential was set sufficiently positive to prevent protamine transfer across the micropipet-supported liquid/liquid interface. At *t* = 0, the tip potential was stepped to a sufficiently negative value to drive diffusion-limited transfer of protamine into the micropipet. The tip current decayed as protamine was depleted at either tip position (solid lines in Figure 4A). At the short distance, protamine was depleted near the NE, inducing dissociation of protamine from the NE (Figure 3B) and enhancing the tip current (*t* < ∼0.5 s). The enhancement of the tip current at the short tip–NE distance was emphasized by plotting the tip current against 1/*t* ^1/2^ (circles in Figure 4A). Eventually, a lower steady-state current was obtained at the short distance, where the NE partially hindered the diffusion of protamine to the tip. The short tip–NE distance, *d*, was obtained from chronoamperogram analysis (see below), and the long distance was determined from the travel distance of the piezo positioner.

**Figure 4:**
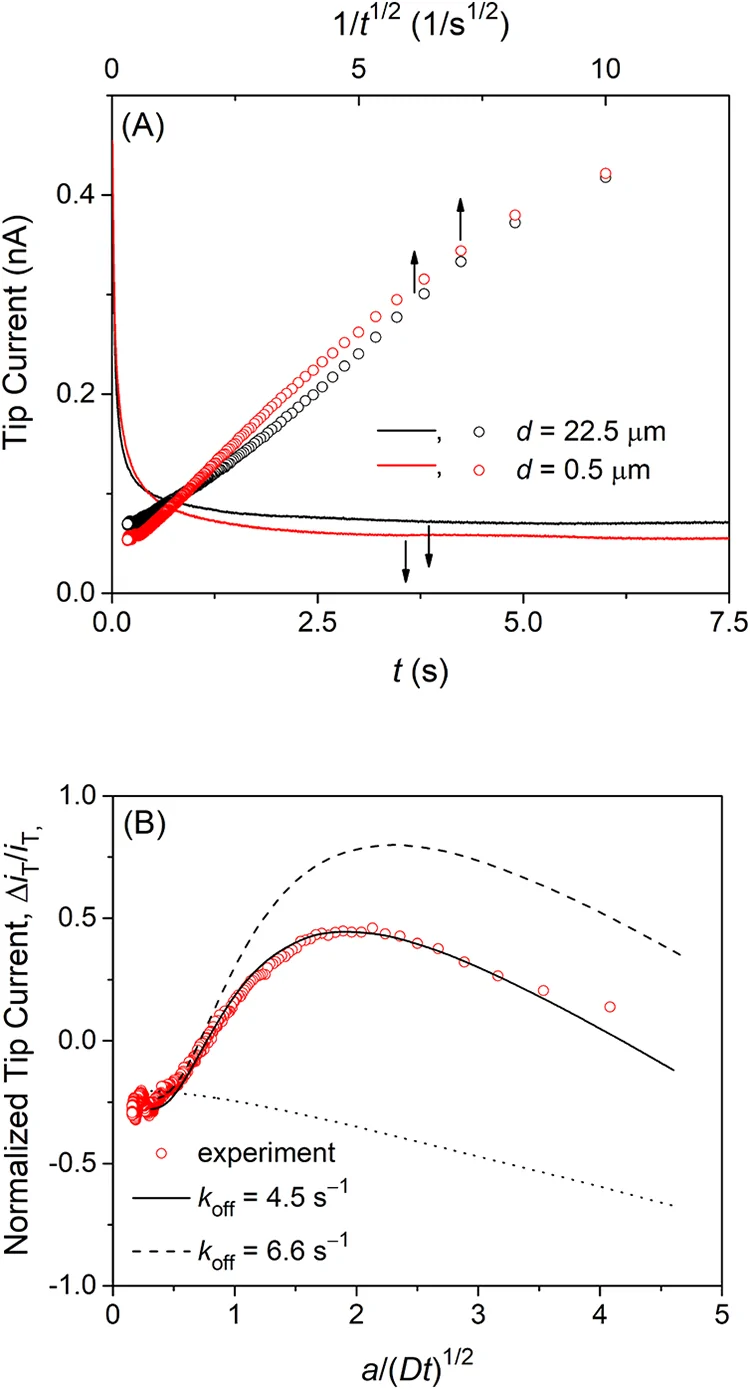
(A) Chronoamperograms of protamine at a 10 μm-diameter micropipet tip positioned far from (black) and near (red) the NE in MIB containing 10 μM protamine and 10 nM nanobody. Sampling interval, 5 ms. The tip–NE distance, *d*, of 0.5 μm was determined by analysis of the chronoamperogram in part (B). (B) Experimental chronoamperogram of protamine after subtraction (circles), compared with the theoretical curve (solid and dashed lines) with β = 1.0 × 10^5^ M^–1^, Γ_s_ = 53 pmol/cm^2^, and *d* = 0.5 μm. The theoretical curve without interactions (dotted line) used Γ_s_ = 0 pmol/cm^2^. Original chronoamperograms are shown in part (A).

Experimental chronoamperograms were analyzed to demonstrate that the nanobody interfered with the dissociative transport of protamine from the NPC but not with the pre-equilibrated association of protamine with the NE. We measured the tip current at short and long distances from the NE to separate the protamine-transfer current from the charging current due to the potential step.^11,43^ The charging current was independent of the tip–substrate distance owing to the high resistance of the organic electrolyte solution. The charging current was eliminated by subtracting a chronoamperogram at the long tip–NE distance from one at the short distance (red circles in Figure 4B). The subtracted experimental current corresponds to the difference between the tip currents attributable to protamine transfer at long and short distances.

A difference in tip currents, Δ*i*_T_, between short and long distances from the NE agreed with the simulated difference (circles and solid line, respectively, in Figure 4B). Specifically, a chronoamperometric tip current, *i*_T_, was simulated for the long distance using the finite element method, yielding an empirical equation as^46^

1 $$\frac{i_{T}}{i_{T,\infty }}=0.6646+\frac{0.3818a}{\sqrt{\mathrm{Dt}}}+0.3354\exp\left(-\frac{0.7057a}{\sqrt{\mathrm{Dt}}}\right)$$

with

2 $$i_{T,\infty }=4\mathrm{xzFDca}$$

where *i*_T,∞_ is the steady-state current at the tip in the bulk solution, *a* is the tip inner radius, *t* is the time after the step in the tip potential, *D* and *c* are the diffusion coefficient and concentration of protamine in the bulk solution, *x* is a function of *RG* (=*r*_g_/*a*, where *r*_g_ is the tip outer radius), and *z* is the charge of a protamine molecule (+20). A chronoamperogram at a short distance was also simulated using the finite element method (see SI). Equation 1 was subtracted from the tip current simulated at the short distance to obtain the theoretical difference.

Fitting the experimental difference to the theoretical difference determined a dissociation rate constant, *k*_off_, an equilibrium association constant, β, and a concentration of binding sites, Γ_s_, for NPC–protamine interactions. Best fits yielded *k*_off_ = (4.4 ± 0.3) s^–1^ (*N* = 4) for fixed β and Γ_s_ values of 1.0 × 10^5^ M^–1^ and 5.3 × 10 pmol/cm^2^, respectively. The β and Γ_s_ values are very similar to those obtained without the nanobody, which does not affect the pre-equilibrated binding of protamine to the NPC. By contrast, the *k*_off_ value was significantly lower than that of 6.6 s^–1^ obtained in the absence of the nanobody.^11^ This result indicates that the nanobody hindered the tip-induced dissociative transport of protamine through the NPC. The β, Γ_s_, and *k*_off_ values without the nanobody were used to simulate a higher tip current response than that observed with 10 nM nanobody. The nanobody–NPC association confirms the presence of Nup98 in the NPC of the NE, which was isolated from the nucleus and supported by the microporous membrane (Figure 3A). The tip current without protamine binding to the NPC was also simulated to represent the expected response when protamine transport was completely blocked. Experimental currents are higher than those without protamine binding to the NPC, thereby indicating that the nanobody kinetically interferes with the dissociative transport of protamine from the NPC by partially clogging the nanopore.

## Effect of Nanobody Concentrations

We observed a decrease in NPC permeability to protamine as nanobody concentration increased, thereby demonstrating NPC–nanobody interactions. As the nanobody concentration was lowered from 10 nM, the normalized tip current after subtraction increased, as represented by 3.2 nM nanobody (Figure 5A). This result indicates that less nanobody was bound to the NPC, thereby reducing the hindrance to the dissociative transport of protamine. By contrast, the normalized tip current after subtraction decreased as the nanobody concentration was increased to 100 nM (Figure 5B). The resultant tip current, however, was still higher than simulated without the binding of protamine to the NPC, thereby indicating that protamine transport was not completely blocked by the nanobody. This result is expected from the local blockage of Nup98 by the nanobody, which does not bind other barrier-forming FG-rich nups.^17^ Moreover, super-resolution fluorescence microscopy revealed that a fluorescence conjugate of the anti-Nup98 nanobody was bound to the periphery of the nanopore,^21^ thereby only partially blocking the nanopore. It should be noted that protamine transport can be blocked by the nanobody not only through steric repulsion but also through electrostatic repulsion. Protamine is highly positively charged, and the nanobody can also be charged, although the charge is unknown.

**Figure 5:**
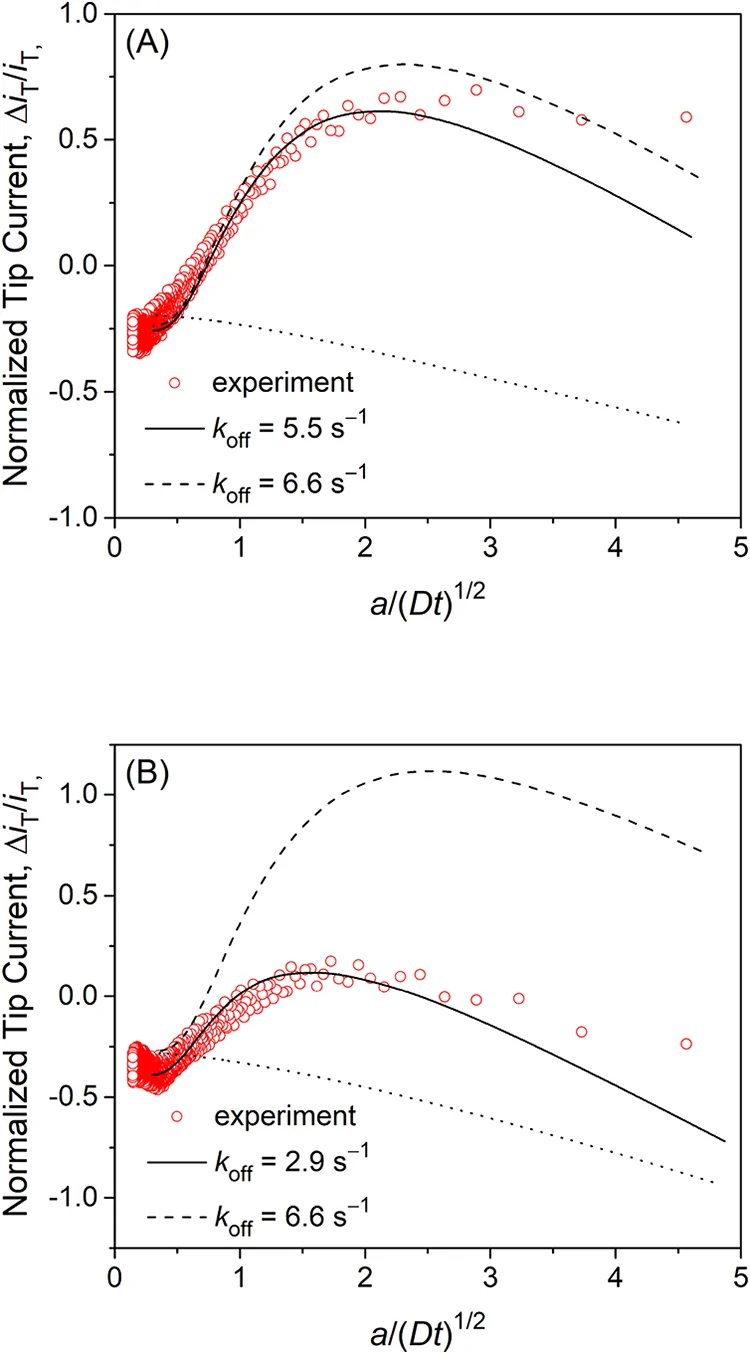
Experimental chronoamperograms of protamine (circles) in the presence of (A) 3.2 and (B) 100 nM nanobody at 10 μm-diameter micropipets, subtracted and compared with theoretical curves (solid and dashed lines) with *d* = (A) 0.5 μm and (B) 0.3 μm, in addition to β = 1.0 × 10^5^ M^–1^, Γ_s_ = 50 pmol/cm^2^. The theoretical curve without interactions (dotted line) employed Γ_s_ = 0 pmol/cm^2^.

An even higher concentration of 320 nM nanobody did not affect the tip CV of protamine during the short potential cycle to ensure the protamine-selective tip current. The tip, however, was gradually fouled by the high-concentration nanobody during tip positioning to reduce the diffusion-limited tip current, thereby hampering reliable SECM measurements. We attribute the tip fouling to nanobody adsorption at the micropipet-supported nitrobenzene/water interface.

The subtracted tip currents at different nanobody concentrations agreed well with the simulated values, demonstrating that the nanobody concentration affects *k*_off_ but not β or Γ_s_. The β and Γ_s_ values were independent of the nanobody concentration because the nanobody does not affect the pre-equilibrated amount of protamine bound to the NPC. By contrast, *k*_off_ decreased with increasing nanobody concentration. A *k*_off_ value of 6.3 ± 0.3 s^–1^ (*N* = 4) at the lowest nanobody concentration of 0.32 nM was similar to that obtained in the absence of the nanobody.^11^ This result indicates that the dissociation constant of the Nup98–nanobody complex was much higher than 0.32 nM. By contrast, similar *k*_off_ values of 3.3 ± 0.4 s^–1^ (*N* = 4) and 2.8 ± 0.1 s^–1^ (*N* = 3) were obtained with nanobody concentrations of 32 and 100 nM, respectively. This result indicates that the dissociation constant of the Nup98–nanobody complex was much lower than 100 nM, at which point the NPC was nearly saturated with the nanobody. The lowest *k*_off_ values are ∼40% of the maximum value as obtained without the nanobody, which selectively binds to Nup98 ^17^ and only partially blocks the NPC.

## Determination of NPC–Nanobody Dissociation Constant

We analyzed the dependence of the NPC permeability on the nanobody concentration to determine the dissociation constant, *K*_D_, for NPC–nanobody interactions. Specifically, we assumed that nanobody binding to the NPC on the NE surface follows a Frumkin isotherm, as represented by

3 $$\frac{\theta_{\mathrm{NB}}}{1-\theta_{\mathrm{NB}}}=\mathrm{RT}\,\ln\frac{c_{\mathrm{NB}}}{K_{D}}+g^{\prime }\theta_{\mathrm{NB}}$$

with

4 $$\theta_{\mathrm{NB}}=\frac{\Gamma_{\mathrm{NB}}}{\Gamma_{\mathrm{nup}}}$$

where *c*_NB_ is the nanobody concentration in the solution, *g*′ is a parameter that captures the heterogeneity of the binding sites, interactions among bound nanobody molecules, or both, and Γ_NB_ and Γ_nup_ represent the fractional and total concentrations of the nanobody and Nup98, respectively. We also assumed that the resulting permeability of the NPC to protamine, *k*_off_, follows

5 $$k_{off}=k_{wo}\left(1-\theta_{\mathrm{NB}}\right)+k_{w}\theta_{\mathrm{NB}}$$

where *k*_wo_ and *k*_w_ are dissociation rate constants when the NPC is free from or saturated with the nanobody, respectively.

The plot of the experimental *k*_off_ values versus the nanobody concentration aligned well with the theoretical plot based on eqs 3 and 5 (Figure 6A). The good fits yielded *K*_D_ = 7.7 nM, *g′* = 0, *k*_wo_ = 6.6 s^–1^, and *k*_w_ = 2.5 s^–1^. The *K*_D_ value was significantly larger than the previously reported value of *K*_D_ = 1.7 nM,^20^ which shifts the theoretical plot away from the experimental one (Figure 6A). The *K*_D_ value of 1.7 nM was determined by biolayer interferometry of isolated Nup98 bound to the nanobody conjugated to an optical sensor surface. We attribute the difference to enthalpic and entropic losses in the free energy required for the nanobody to enter the condensed barrier phase to interact with Nup98.^47^ Enthalpically, the hydrophilic nanobody must interact with the hydrophobic environment of the FG-rich barrier phase. Entropically, the nanobody must restrict the conformation of highly disordered FG-rich domains. More quantitatively, the partition of the nanobody (NB) into the transport barrier of the NPC is followed by the binding of the nanobody to Nup98, as given by

6 NB(aq)⇌NB(NPC)

7 NB(NPC)+Nup98(NPC)⇌NB−Nup98(NPC)

to yield

$$K_{D}=\frac{{K^{\prime }}_{D}}{K_{p}}$$ 8

where *K*_p_ is the partition coefficient of eq 6 and *K*_D^′^_ is the dissociation constant of eq 7. We assume that *K* ^′^_D_ is equivalent to a *K*_D_ of 1.7 nM,^20^ as determined by biolayer interferometry, to estimate *K*_p_ = 0.22 from eq 8 with *K*_D_ = 7.7 nM. This partition coefficient is very similar to that of GFP (28 kDa) between water and the hydrogel of FG-rich *Xenopus* nups (0.11–0.33).^9^ The similarity is interesting but is likely coincidental, as the nanobody and GFP differ in molecular size and physicochemical properties. It should be noted that transient SECM required the binding of FG units to protamine, which may affect the chemical environment of the condensate and, subsequently, the partition coefficient.

**Figure 6:**
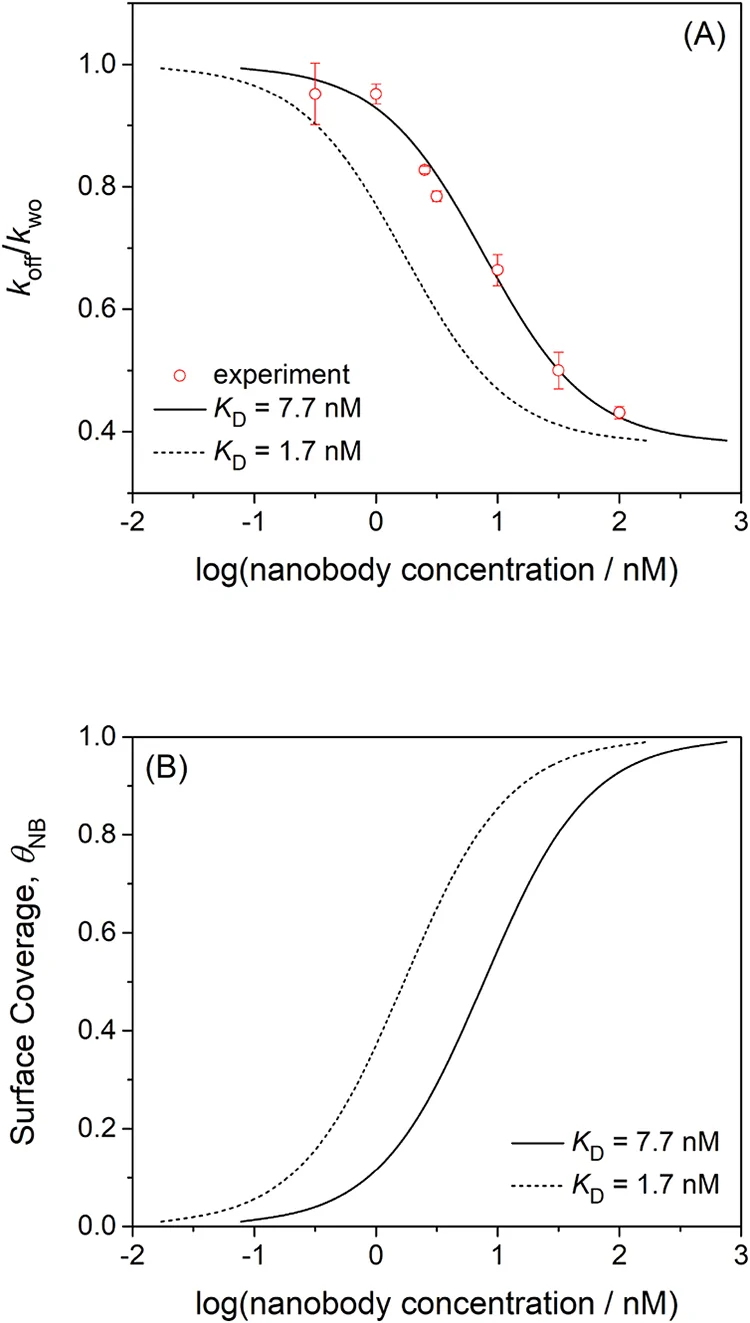
(A) Experimental permeability of protamine with different concentrations of nanobody as compared with permeability calculated by using eqs 1 and 3 with *g*′ = 0. (B) Calculated fractions of the nanobody bound to Nup98 as the binding sites of the NPC.

Our SECM method allows determination of θ_NB_ as nearly saturated at 100 nM nanobody (Figure 6B) but not of Γ_nup_ or Γ_NB_ because only θ_NB_ appears in eqs 3 and 5. Instead, we estimated a Γ_nup_ value from structural information on Nup98 in the NPC of the *Xenopus* oocyte nucleus. Specifically, we estimated Γ_nup_ = 1.92 × 10^3^ Nup98/μm^2^ based on 48 copies of Nup98 per NPC^48^ and a surface concentration of 40 NPC/μm^2^.^49^ The concentration of Nup98 is not high enough to cause interactions among nanobodies, thereby yielding *g′* = 0. This *g′* value also implies a homogeneous nanobody binding site, as expected for the C-terminal globular domain of Nup98 (Figure 1B).^21^

It should be emphasized that the Frumkin isotherm with *g′* = 0 (Figure 6A) is equivalent to the Langmuir isotherm, supporting the conclusion that the nanobody–NPC interactions are specific. The Langmuir isotherm assumes homogeneous adsorption sites, which, in this case, should be specific because the nanobody can interact specifically with Nup98 (Figure 1). The coexistence of nonspecific adsorption sites, or competition between specific and nonspecific adsorption at the same sites, results in a deviation from the Langmuir isotherm.^50^ The dominance of specific adsorption also excludes steric or general blockage of the nanopore by the small nanobody, which can partition into the transport barrier without any interactions (i.e., based only on eq 6).

## A Comparison with Steady-State SECM

Transient SECM yielded a significantly larger tip current response than the steady-state counterpart under our experimental conditions, thereby enabling precise measurement of protamine transport rates with various nanobody concentrations (Figure 6A). A change in normalized tip current of ∼*i*_T,∞_ was simulated as the nanobody concentration increased to saturate the NPC with the nanobody (Figure 7A). This result is consistent with experimental values (Figures 5B, 6A,B), which deviate from theoretical values owing to a noise amplitude of ±0.1*i*_T,∞_ to estimate a signal-to-noise ratio of ∼*i*_T,∞_/0.1*i*_T,∞_ = ∼10. The difference in tip current between the NPC free of and saturated with the nanobody decreases over longer times, reaching a nearly steady value of ∼0.1*i*_T,∞_. This result is consistent with simulations of the steady-state tip current at various distances between the tip and the NE, with the NPC either free of or saturated with the nanobody (Figure 7B).

**Figure 7:**
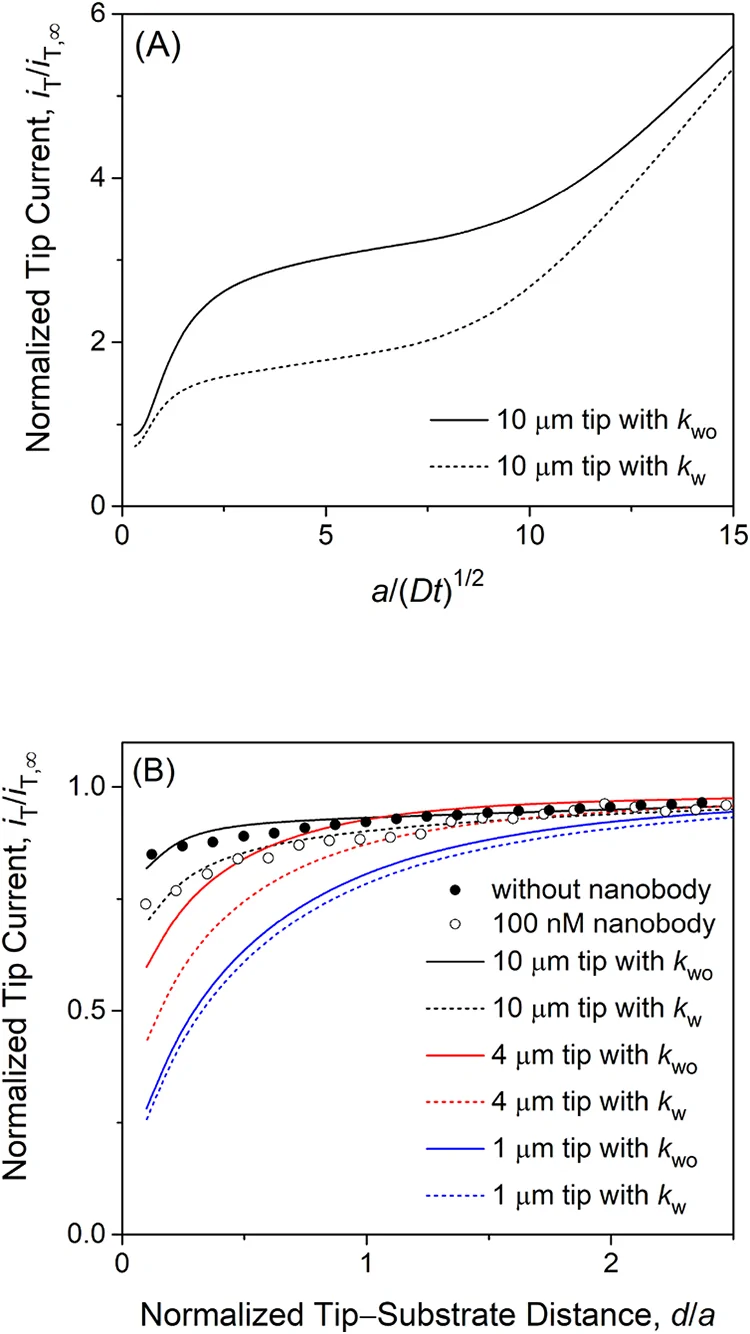
(A) Chronoamperograms and (B) steady-state approach curves as simulated for dissociative protamine transport through NPCs that are free from (solid lines) or saturated with (dotted lines) the nanobody with *k*_wo_ = 6.6 s^–1^ and *k*_w_ = 2.5 s^–1^, respectively. Circles in part (B) represent experimental approach curves.

We simulated approach curves based on the steady-state current response of tips with various diameters and found that a 4 μm-diameter tip is most sensitive to NPC blockage by the nanobody (Figure 7B). The simulated approach curves were validated for a 10 μm-diameter tip to match experimental curves when the NPC was either free of or saturated with the nanobody. The corresponding difference in the tip current with and without the nanobody was only ∼0.12*i*_T,∞_ at the normalized distance of 0.1. The experimental tip currents at the shortest distance deviated from theoretical curves by ∼0.04*i*_T,∞_, which we treated as noise to estimate a signal-to-noise ratio of ∼0.12*i*_T,∞_/∼0.04*i*_T,∞_ = ∼3. This value is significantly lower than ∼10, as estimated for transient measurements above. The difference increased and reached a maximum when the tip diameter was reduced to 4 μm. The maximum difference, however, was still ∼0.17*i*_T,∞_. A smaller 1 μm-diameter tip yielded a smaller difference in the normalized tip current between with and without the nanobody, as indicated by a nearly diminishing effect of the nanobody. In contrast to the steady-state case, our simulation demonstrates that a 4 μm-diameter pipet yields a smaller difference of ∼0.5*i*_T,∞_ in transient tip current (Figure S2) than a 10 μm-diameter pipet (Figure 7A).

## Conclusions

In this work, we demonstrated a new application of transient SECM to measure protein–protein interactions by investigating the permeability of the nanobody-bound NPC to a polycationic peptide. A nanobody entered the transport barrier condensed within the NPC, bound to Nup98, and partially blocked the nanopore, thereby hindering the transport of the polypeptide probe. Transient SECM detected a small change in the permeability of the partially blocked NPC with greater sensitivity than the steady-state counterpart. We obtained a dissociation constant of 7.7 nM, which is significantly larger than that of 1.7 nM measured with a surface-conjugated nanobody and Nup98 isolated in solution.^20^ This quantitative comparison indicates that partitioning a nanobody into hydrophobic, disordered biomolecular condensates of FG-rich nups in the authentic NPC is hindered both enthalpically and entropically. Previously, protein–protein^26,27,29^ and DNA–protein^28^ interactions were detected by steady-state SECM but were not quantitatively measured. Our transient method will be applicable to various NPC-binding proteins, including nuclear transport receptors and their cargo complexes, which play crucial roles in regulating gene expression and the cell cycle.

Our transient SECM method offers unique advantages that complement other analytical methods for quantitative studies of protein interactions with the NPC. Importantly, FG-rich nups form biomolecular condensates that create a hydrophobic environment, thereby affecting NPC–protein interactions. The SECM method does not require conjugating target proteins to fluorophores, which can significantly interact with FG-nup condensates.^9,31^ SECM enables the study of FG-rich nups condensed within the authentic NPC, rather than isolated ones with markedly different protein–protein interaction strengths.^30^ Moreover, the SECM method can correlate protein-binding events with NPC permeability, thereby enabling an anti-nup nanobody to indicate whether the target nup contributes to transport. By contrast, the SECM method is not sensitive to the binding of a nanobody to a nup that does not contribute to NPC-mediated molecular transport. The anticipated binding of the nanobody to the NPC can be ensured, for instance, by super-resolution fluorescence microscopy^51^ to determine the roles of nups as transport barriers or structural frameworks.^52^ More broadly, SECM and fluorescence methods are complementary and will be useful for cross-checking the effects of an electrochemical probe and a fluorescence label on protein–protein interactions.

## Supplementary Material



## Notes

Expression and purification of nanobody, finite element simulation (PDF)

## †

Department of Chemistry, University of Illinois at Urbana–Champaign, Urbana, Illinois 61801, United States

## References

[1] Petrovic S., Mobbs G. W., Hoelz A. (2026). Structure, function and assembly
of nuclear pore complexes. Nat. Rev. Mol. Cell
Biol..

[2] Yu W., Rush C., Tingey M., Junod S., Yang W. (2023). Application
of super-resolution speed microscopy in the study of cellular dynamics. Chem. Biomed. Imaging.

[3] Mobbs G. W., Petrovic S., Hoelz A. (2026). Nucleocytoplasmic transport. Annu. Rev. Biochem..

[4] Simon, D. N. ; Rout, M. P. Cancer and the nuclear pore complex. In Cancer biology and the nuclear envelope: Recent advances may elucidate past paradoxes; Schirmer, E. C. ; DeLasHeras, J. I. , Eds.; Springer: New York, NY, 2014; Vol. 773, pp 285–307. 10.1007/978-1-4899-8032-8_13 24563353

[5] Coyne A. N., Rothstein J. D. (2022). Nuclear
pore complexes—a doorway to neural injury
in neurodegeneration. Nat. Rev. Neurol..

[6] Chien C.-Y., Mobbs G. W., Ehrenkranz J., Hoelz A. (2025). Viral interference
of nucleocytoplasmic transport. J. Biol. Chem..

[7] Timney B. L., Raveh B., Mironska R., Trivedi J. M., Kim S. J., Russel D., Wente S. R., Sali A., Rout M. P. (2016). Simple
rules for passive diffusion through the nuclear pore complex. J. Cell Biol..

[8] Kehlenbach R. H., Neumann P., Ficner R., Dickmanns A. (2023). Interaction
of nucleoporins with nuclear transport receptors: A structural perspective. Biol. Chem..

[9] Frey S., Rees R., Schunemann J., Ng S. C., Funfgeld K., Huyton T., Görlich D. (2018). Surface properties determining passage
rates of proteins through nuclear pores. Cell.

[10] Shi K. Y., Mori E., Nizami Z. F., Lin Y., Kato M., Xiang S., Wu L. C., Ding M., Yu Y., Gall J. G., McKnight S. L. (2017). Toxic PR_n_ poly-dipeptides
encoded by the *C9orf72* repeat expansion block nuclear
import and export. Proc. Natl. Acad. Sci. U.S.A..

[11] Huang S.-H., Parandhaman M., Jyothi Ravi M., Janda D. C., Amemiya S. (2024). Nanoscale
interactions of arginine-containing dipeptide repeats with nuclear
pore complexes as measured by transient scanning electrochemical microscopy. Chem. Sci..

[12] Huang S.-H., Parandhaman M., Jyothi Ravi M., Janda D. C., Amemiya S. (2025). Nanoscale
hydrophobicity of transport barriers in the nuclear pore complex as
compared with the liquid/liquid interface by scanning electrochemical
microscopy. Anal. Chem..

[13] Amemiya S., Bard A. J., Fan F.-R. F., Mirkin M. V., Unwin P. R. (2008). Scanning
electrochemical microscopy. Annu. Rev. Anal.
Chem..

[14] Shen, M. ; Amemiya, S. Molecular transport in membranes. In Scanning Electrochemical Microscopy, 3rd ed.; Bard, A. J. ; Mirkin, M. V. , Eds.; CRC Press: Boca Raton, FL, 2022; pp 181–205.

[15] Putnam S. T., Santiago-Carboney A., Qian P., Rodríguez-López J. (2025). Scanning electrochemical
microscopy: An evolving toolbox for revealing the chemistry within
electrochemical processes. Anal. Chem..

[16] Muyldermans S. (2013). Nanobodies:
Natural single-domain antibodies. Annu. Rev.
Biochem..

[17] Pardon E., Laeremans T., Triest S., Rasmussen S. G. F., Wohlkonig A., Ruf A., Muyldermans S., Hol W. G. J., Kobilka B. K., Steyaert J. (2014). A general protocol
for the generation of nanobodies for structural biology. Nat. Protoc..

[18] Chug H., Trakhanov S., Hulsmann B. B., Pleiner T., Görlich D. (2015). Crystal structure
of the metazoan Nup62·Nup58·Nup54 nucleoporin complex. Science.

[19] Göttfert F., Pleiner T., Heine J., Westphal V., Görlich D., Sahl S. J., Hell S. W. (2017). Strong signal increase in STED fluorescence
microscopy by imaging regions of subdiffraction extent. Proc. Natl. Acad. Sci. U.S.A..

[20] Colom M. S., Fu Z., Gunkel P., Güttler T., Trakhanov S., Srinivasan V., Gregor K., Pleiner T., Görlich D. (2024). A checkpoint
function for Nup98 in nuclear pore formation suggested by novel inhibitory
nanobodies. EMBO J..

[21] Pleiner T., Bates M., Trakhanov S., Lee C. T., Schliep J. E., Chug H., Bohning M., Stark H., Urlaub H., Görlich D. (2015). Nanobodies:
Site-specific labeling for super-resolution
imaging, rapid epitope-mapping and native protein complex isolation. Elife.

[22] Huang S.-H., Amemiya S. (2024). Transient theory for
scanning electrochemical microscopy
of biological membrane transport: Uncovering membrane–permeant
interactions. Analyst.

[23] Guo J., Amemiya S. (2005). Permeability of the
nuclear envelope at isolated *xenopus* oocyte nuclei
studied by scanning electrochemical
microscopy. Anal. Chem..

[24] Kim J., Izadyar A., Nioradze N., Amemiya S. (2013). Nanoscale mechanism
of molecular transport through the nuclear pore complex as studied
by scanning electrochemical microscopy. J. Am.
Chem. Soc..

[25] Amemiya S. (2021). Nanoelectrochemical
study of molecular transport through the nuclear pore complex. Chem. Rec..

[26] Shiku H., Matsue T., Uchida I. (1996). Detection of microspotted carcinoembryonic
antigen on a glass substrate by scanning electrochemical microscopy. Anal. Chem..

[27] Wittstock G., Yu K.-j., Halsall H. B., Ridgway T. H., Heineman W. R. (1995). Imaging
of immobilized antibody layers with scanning electrochemical microscopy. Anal. Chem..

[28] Gorodetsky A. A., Hammond W. J., Hill M. G., Slowinski K., Barton J. K. (2008). Scanning electrochemical microscopy of DNA monolayers
modified with Nile Blue. Langmuir.

[29] Chen R., Pathirathna P., Balla R. J., Kim J., Amemiya S. (2024). Nanoscale
quantitative imaging of single nuclear pore complexes by scanning
electrochemical microscopy. Anal. Chem..

[30] Hayama R., Sorci M., Keating
Iv J. J., Hecht L. M., Plawsky J. L., Belfort G., Chait B. T., Rout M. P. (2019). Interactions of
nuclear transport factors and surface-conjugated FG nucleoporins:
Insights and limitations. PLoS One.

[31] Ng S. C., Güttler T., Görlich D. (2021). Recapitulation of selective nuclear
import and export with a perfectly repeated 12mer GLFG peptide. Nat. Commun..

[32] Yuan Y., Amemiya S. (2004). Facilitated protamine
transfer at polarized water/1,2-dichloroethane
interfaces studied by cyclic voltammetry and chronoamperometry at
micropipet electrodes. Anal. Chem..

[33] Guo J., Amemiya S. (2006). Voltammetric heparin-selective
electrode based on thin
liquid membrane with conducting polymer-modified solid support. Anal. Chem..

[34] Frey S., Görlich D. (2014). A new set of highly efficient, tag-cleaving proteases
for purifying recombinant proteins. J. Chromatogr.
A.

[35] Pathirathna P., Balla R. J., Meng G., Wei Z., Amemiya S. (2019). Nanoscale
electrostatic gating of molecular transport through nuclear pore complexes
as probed by scanning electrochemical microscopy. Chem. Sci..

[36] Nioradze N., Kim J., Amemiya S. (2011). Quasi-steady-state
voltammetry of rapid electron transfer
reactions at the macroscopic substrate of the scanning electrochemical
microscope. Anal. Chem..

[37] Jantz D. T., Balla R. J., Huang S.-H., Kurapati N., Amemiya S., Leonard K. C. (2021). Simultaneous intelligent
imaging of nanoscale reactivity
and topography by scanning electrochemical microscopy. Anal. Chem..

[38] Ishimatsu R., Kim J., Jing P., Striemer C. C., Fang D. Z., Fauchet P. M., McGrath J. L., Amemiya S. (2010). Ion-selective
permeability of a ultrathin
nanopore silicon membrane as probed by scanning electrochemical microscopy
using micropipet-supported ities tips. Anal.
Chem..

[39] Kim J., Izadyar A., Shen M., Ishimatsu R., Amemiya S. (2014). Ion permeability of the nuclear pore
complex and ion-induced
macromolecular permeation as studied by scanning electrochemical and
fluorescence microscopy. Anal. Chem..

[40] Chen R., Balla R. J., Lima A., Amemiya S. (2017). Characterization of
nanopipet-supported ITIES tips for scanning electrochemical microscopy
of single solid-state nanopores. Anal. Chem..

[41] Pathirathna P., Balla R. J., Jantz D. T., Kurapati N., Gramm E. R., Leonard K. C., Amemiya S. (2019). Probing high
permeability of nuclear
pore complexes by scanning electrochemical microscopy: Ca^2+^ effects on transport barriers. Anal. Chem..

[42] Jäggi R. D., Franco-Obregon A., Muhlhausser P., Thomas F., Kutay U., Ensslin K. (2003). Modulation
of nuclear pore topology by transport modifiers. Biophys. J..

[43] Slevin C. J., Liljeroth P., Kontturi K. (2003). Measurement of the adsorption of
drug ions at model membranes by scanning electrochemical microscopy. Langmuir.

[44] Meyerhoff M. E., Fu B., Bakker E., Yun J.-H., Yang V. C. (1996). Polyion-sensitive
membrane electrodes for biomedical analysis. Anal. Chem..

[45] Wang X., Sun M., Ferguson S. A., Hoff J. D., Qin Y., Bailey R. C., Meyerhoff M. E. (2019). Ionophore-based
biphasic chemical sensing in droplet
microfluidics. Angew. Chem., Int. Ed..

[46] Yuan Y., Wang L., Amemiya S. (2004). Chronoamperometry
at micropipet electrodes
for determination of diffusion coefficients and transferred charges
at liquid/liquid interfaces. Anal. Chem..

[47] Rush C., Jiang Z., Tingey M., Feng F., Yang W. (2023). Unveiling
the complexity: Assessing models describing the structure and function
of the nuclear pore complex. Front. Cell Dev.
Biol..

[48] Ori A., Banterle N., Iskar M., Andrés-Pons A., Escher C., Bui H. K., Sparks L., Solis-Mezarino V., Rinner O., Bork P., Lemke E. A., Beck M. (2013). Cell type-specific
nuclear pores: A case in point for context-dependent stoichiometry
of molecular machines. Mol. Syst. Biol..

[49] Wang H., Clapham D. E. (1999). Conformational changes of the in situ nuclear pore
complex. Biophys. J..

[50] Frutiger A., Tanno A., Hwu S., Tiefenauer R. F., Vörös J., Nakatsuka N. (2021). Nonspecific
binding—Fundamental
concepts and consequences for biosensing applications. Chem. Rev..

[51] Sau A., Schnorrenberg S., Huang Z., Bandyopadhyay D., Sharma A., Gürth C.-M., Dave S., Musser S. M. (2025). Overlapping
nuclear import and export paths unveiled by two-colour MINFLUX. Nature.

[52] Yang W. D. (2011). 'Natively
unfolded' nucleoporins in nucleocytoplasmic transport clustered
or
evenly distributed?. Nucleus.

