# Supplementary material for: Permeability-Based Measurement of Nanobody Binding to Nuclear Pore Complexes by Transient Scanning Electrochemical Microscopy
Source: Anal Chem. 2026 Sep 24;98(41):29639–47. doi: 10.1021/acs.analchem.6c04319 (PMC13689402; doi:10.1021/acs.analchem.6c04319)
Supplement: Supplementary file 1 [file ac6c04319_si_001.pdf]

Supporting Information

Permeability-Based Measurement of Nanobody Binding to Nuclear Pore Complexes by Transient Scanning Electrochemical Microscopy

Moghitha Parandhaman, Siao-Han Huang, Kathryn G. Dzurik, W. Seth Childers, and Shigeru Amemiya*

Department of Chemistry, University of Pittsburgh, 219 Parkman Avenue, Pittsburgh, Pennsylvania, 15260, United States

* To whom correspondence should be addressed. E-mail: amemiya@pitt.edu. Fax: 412-624-8611.

Contents:

Expression and Purification of Nanobody S-2

Finite Element Simulation S-6

Transient SECM Simulation of 4 μm Tip S-10

References S-11

Expression and Purification of xNup98-Nb1t (+1Cys). The xNup98-Nb1t (+1Cys) nanobody was expressed and purified as previously reported.[S-1] An expression plasmid, pTP443 (Addgene 216361), was transformed into *E. coli* Rosetta (DE3) competent cells and grown in Lysogeny Broth (LB) Miller medium supplemented with kanamycin (30 µg/mL) and chloramphenicol (30 µg/mL) for an overnight at 37 °C. The overnight culture was diluted 1:100 into fresh LB medium containing kanamycin and chloramphenicol. The cells were grown to mid-log phase at 30 °C and induced with 200 µM IPTG overnight at 25 °C. Cells were collected by centrifugation at 4,122 × *g* for 10 min at 4 °C. Pellets were resuspended in buffer (300 mM NaCl, 50 mM Tris-Cl pH 7.4, 20 mM imidazole, and 1 mM DTT) and centrifuged at 4,122 × *g* for 10 min at 4 °C. Pellets were stored at –80 °C until purification.

The cell pellets were thawed on ice and resuspended in lysis buffer (290 mM NaCl, 45 mM Tris-Cl, pH 7.5, 4.5 mM $MgCl_2$, 15 mM imidazole, 10 mM DTT, 100 U DNase I, and a protease inhibitor). The inhibitor was obtained as a Pierce EDTA-free tablet. The resuspended cells were lysed using a high-pressure homogenizer (Emulsiflex-C3, Avestin, Ottawa, ON, Canada). Specifically, the solution was equilibrated at 4 °C for 15 minutes without applied pressure and then processed at 15,000 psi for 45 minutes to achieve cell lysis. The whole-cell lysate was centrifuged at 20,000 × *g* for 45 min at 4 °C to separate soluble proteins containing the His-tagged xNup98-Nb1t (+1Cys) nanobody from insoluble cell debris and aggregates in the pellet fraction.

The soluble portion was rocked with a slurry of loose Ni-NTA resin (HisPur, Thermo Fisher Scientific, Waltham, MA) in a wash buffer (290 mM NaCl, 45 mM Tris-Cl pH 7.5, 4.5 mM $MgCl_2$, 15 mM imidazole, and 10 mM DTT) for 2 hours at 4 °C to capture the His-tagged nanobody. The slurry was centrifuged at 4 °C for 2 min at 1,200 × *g* and washed with 25 mL of the wash buffer four times. A disposable 10 mL column was packed with the Ni-NTA resin slurry, and the 14xHis-tagged xNup98-

Nb1t (+1Cys) nanobody was eluted with 10 mL of an elution buffer (290 mM NaCl, 45 mM Tris-Cl pH 7.5, 4.5 mM $MgCl_2$, 10 mM DTT, and 400 mM imidazole).

One unit of SUMO protease (pDG02583, Addgene 104129) was used to completely cleave the His-tag on the xNup98-Nb1t (+1Cys) nanobody for 1 hour at 30 °C and overnight at 4 °C. To remove residual His-tagged nanobody, the protease-treated solution of tagless xNup98-Nb1t (+1Cys) nanobody was incubated and rocked with excess loose Ni resin in dialysis buffer (290 mM NaCl, 45 mM Tris-Cl pH 7.5, 4.5 mM $MgCl_2$, and 10 mM DTT) for an additional 2 hours at 4 °C, then centrifuged at 1,200 × *g* for 2 min at 4 °C and washed 4 times with the dialysis buffer to separate the xNup98-Nb1t (+1Cys) nanobody solution from the resin that captured the uncleaved His-tagged nanobody. The Bradford assay was used to confirm the presence of the nanobody in the separated solution. The nanobody solution was incubated and rocked with a 4-fold excess of loose Ni-NTA resin for 2 hours at 4 °C, then centrifuged at 1,200 × *g* for 2 min at 4 °C to separate the nanobody solution from the resin. Nanodrop (Thermo Fisher Scientific) was used to calculate the nanobody concentration using the molar absorptivity (2.395×10^4 $M^{-1} \cdot cm^{-1}$). The molar absorptivity was calculated using ProtParam. The calculated concentration was 0.556 mg of nanobody per 1 L of culture. SDS-PAGE also confirmed the 100% band purity of the xNup98-Nb1t (+1Cys) nanobody (Figure 2).

Expression and Purification of NEDD8-specific protease 1 (NEDP1). The SUMO protease NEDD8-specific protease 1 (NEDP1) was expressed and purified as previously reported[S-2] by transforming *E. coli* BL21 (DE3) competent cells with plasmid pDG02583 (Addgene 104129). Cells were grown in LB medium with kanamycin (30 μg/mL) at 37 °C overnight. The overnight culture was diluted 100-fold in fresh LB medium containing kanamycin and shaken to mid-log phase. We added 200 μM IPTG to induce NEDP1 expression for 4 hours at 28 °C. We added 1 mM PMSF and 5 mM EDTA to the culture, then shook for 15 minutes. The cells were centrifuged at 4,122 × *g* for 10 minutes at 4 °C.

The resulting cell pellet was resuspended in a buffer containing 300 mM NaCl, 50 mM Tris-Cl (pH 7.5), 20 mM imidazole, and 2 mM DTT. The resuspended cells were repelleted and stored at –80 °C until purification.

The cell pellets were thawed on ice and resuspended in a lysis buffer containing 300 mM NaCl, 50 mM Tris-Cl pH 8.0, 25 mM imidazole, 10 mM DTT, 250 mM sucrose, 100 U DNase I, and a protease inhibitor (Pierce EDTA-free tablet). The resuspended cells were lysed using a high-pressure homogenizer. The solution was equilibrated at 4 °C for 15 minutes without applied pressure, then processed at 15,000 psi for 45 minutes to achieve cell lysis. The whole-cell lysate was centrifuged at 20,000 × *g* for 45 min at 4 °C to separate soluble proteins containing NEDP1 from insoluble proteins and aggregates in the pellet. The soluble protein fraction was applied to an equilibrated HisTrap FF Ni^{2+} Sepharose column (buffer: 300 mM NaCl, 50 mM Tris-Cl pH 8.0, 25 mM imidazole pH 7.5, 250 mM sucrose, and 10 mM DTT) using an FPLC instrument (Äkta Pure 25L, Cytiva, Wilmington, DE) and eluted with this buffer at a flow rate of 5 mL per minute. The histidine-tagged protein was washed four times before elution with elution buffer (300 mM NaCl, 50 mM Tris-Cl pH 8.0, 500 mM imidazole, 250 mM sucrose, and 10 mM DTT).

Table S1. Plasmid Construction

Name	Description	Gene/Insert Name	Vector	Digest Site	Source
pDG02583	14x His, Maltose binding protein, SUMO cleavage site (*Brachypodium distachyon*)	NEDD8-specific protease 1 (NEDP1)	pQE80 derivative	SUMO	RRID:Addgene_104129
pTP443	14x His-NEDD8	xNup98-Nb1t (+1Cys)	pDG-Kan	SUMO	RRID:Addgene_216361

Table S2. Strain Collection

Strain Name	Genotype	Plasmid	Source
	E. coli BL21(DE3)		Invitrogen
	E. coli Rosetta(DE3)		Sigma Aldrich
WSC1830	*E. coli* DH5α	pDG02583	RRID:Addgene_104129
WSC1831	*E. coli* DH5α	pTP443	RRID:Addgene_216361
WSC1835	*E. coli* BL21(DE3)	pDG02583	This work
WSC1838	*E. coli* Rosetta(DE3)	pTP443	This work

Finite Element Simulation. In this work, NPC–protamine interactions are represented by a Langmuir isotherm and implemented in the SECM model based on the homogeneous NE for finite element simulation, as reported previously.[S-3-S-5] Protamine is transported uniformly throughout the entire NE in the hypothetical homogeneous model. In this model, a protamine molecule, P, is transported throughout the entire NE as given by

$$P(w_C) + NE \rightleftharpoons P\text{–}NE \rightleftharpoons P(w_N) + NE \qquad \text{(S1)}$$

where w_C and w_N indicate the solution near the cytoplasmic and nuclear sides of the NE, respectively, and P–NE is the protamine molecule associated with the NE. The rates of the first and second steps, v_C and v_N, in eq S1 are given by

$$v_C = k_{on}c_C(\Gamma_s - \Gamma_P) - k_{off}\Gamma_P \qquad \text{(S2)}$$

$$v_N = k_{off}\Gamma_P - k_{on}c_N(\Gamma_s - \Gamma_P) \qquad \text{(S3)}$$

where k_{on} and k_{off} are the association and dissociation rate constants for protamine binding to the NE, respectively, c_C and c_N are the protamine concentrations on the cytoplasmic and nuclear sides of the NE, respectively, and Γ_s and Γ_P are the concentrations of the interaction site in the NE and of its complex with protamine, respectively. Eqs S2 and S3 are equivalent to the Langmuir isotherm under equilibrium

$$\beta c_i = \frac{\Gamma_P}{\Gamma_s - \Gamma_P} \qquad \text{(S4)}$$

with

$$\beta = \frac{k_{on}}{k_{off}} \qquad \text{(S5)}$$

where i = C or N.

We simulated the diffusion-limited current at a disk-shaped SECM tip, i_T, by solving the axisymmetric (2D) diffusion problem in cylindrical coordinates (Figure S1). The coordinate origin was

set at the NE under the tip center. Initially, the solution phase contained protamine at a bulk concentration c_0 on both the nuclear and cytoplasmic sides of the NE. The time-dependent diffusion of protamine in each solution was described by

$$\frac{\partial c_{\mathrm{i}}}{\partial t} = D\left(\frac{\partial^2 c_{\mathrm{i}}}{\partial r^2} + \frac{1}{r}\frac{\partial c_{\mathrm{i}}}{\partial r} + \frac{\partial^2 c_{\mathrm{i}}}{\partial z^2}\right) \tag{S6}$$

where c_{i} is the protamine concentration at (r, z), D is the diffusion coefficient of protamine, and t is time after the step in the tip potential in chronoamperometry. The left-hand side of eq S6 was set to zero for simulating steady-state approach curves. Eq S6 was solved using the following initial and boundary conditions. Initial conditions at $t = 0$ are given for any location in the bulk solutions as

$$c_{\mathrm{C}}(t = 0) = c_{\mathrm{N}}(t = 0) = c_0 \tag{S7}$$

Accordingly, the entire membrane is initially equilibrated with protamine to yield an initial condition given by eq S4 as

$$\Gamma_{\mathrm{P}}(t=0) = \frac{\beta c_0 \Gamma_{\mathrm{S}}}{1+\beta c_0} \tag{S8}$$

For $t > 0$, protamine is transferred across the micropipet-supported liquid/liquid interface at the diffusion-limited rate, yielding a boundary condition at the interface as

$$c_{\mathrm{C}}(t > 0) = 0 \tag{S9}$$

The depletion of protamine at the tip induces a flux of protamine across the NE under the tip. The corresponding boundary conditions on the cytoplasmic and nuclear sides of the NE are given by

$$D\left(\frac{\partial c_{\mathrm{i}}}{\partial z}\right) = v_{\mathrm{i}} \tag{S10}$$

where v_{C} and v_{N} are defined by eqs S2 and S3, respectively. In addition, the association and dissociation of protamine with or from the NE are induced to yield a boundary condition as

$$D\left(\frac{\partial \Gamma_P}{\partial t}\right) = v_C + v_N \qquad \text{(S11)}$$

Other boundary conditions are given in Figure S-1.

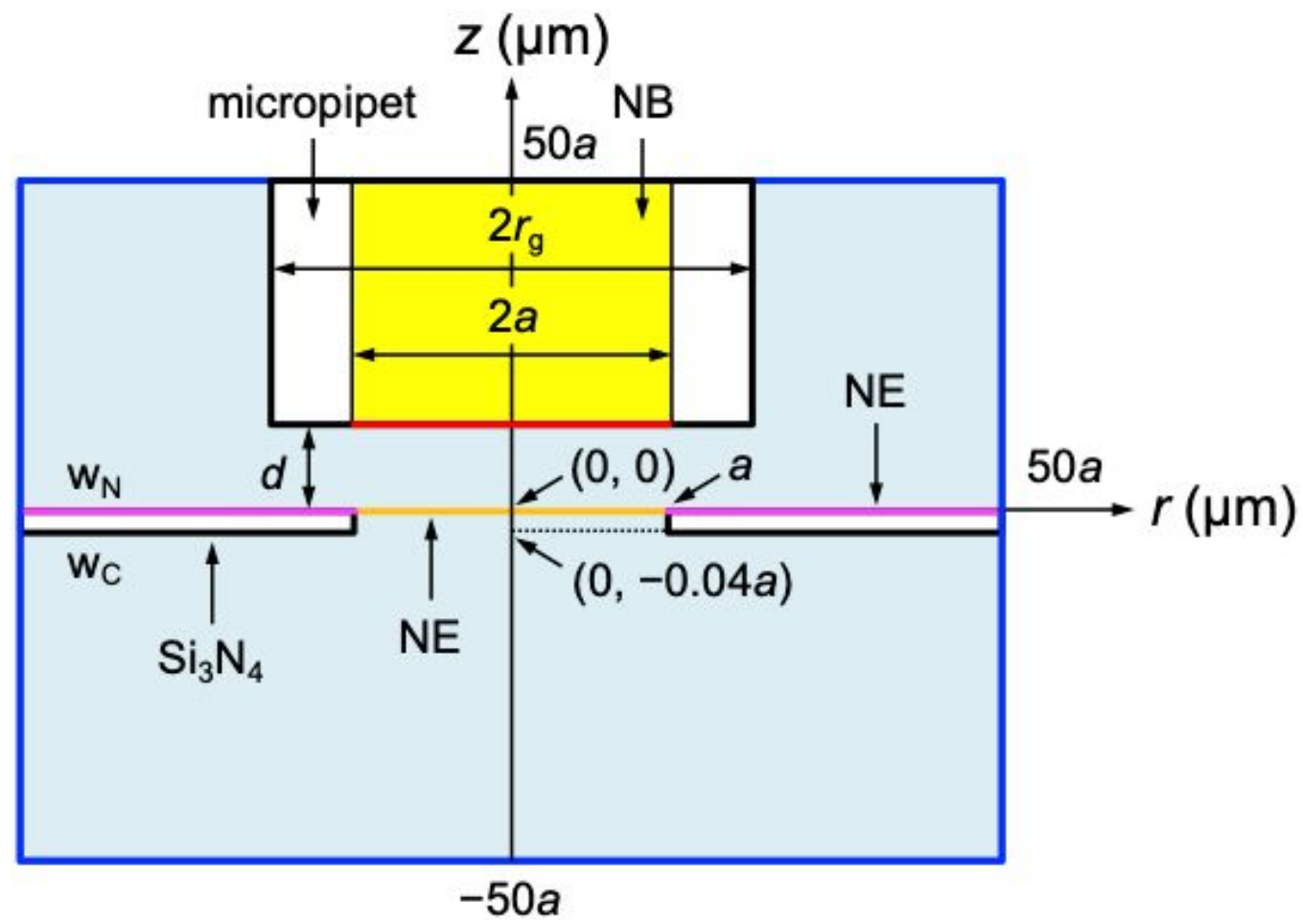

Figure S1. SECM diffusion problem for NPC-mediated protamine transport in cylindrical coordinates. Boundaries are indicated by thick solid lines. Boundary conditions at the tip (red line) and the NE (orange line) are given in the text. There is no normal flux at the symmetry *z-axis* and at the insulating surfaces (black lines), as well as at the NE surrounding the micropore (magenta line). Simulation space limits are represented by blue lines.

The current response of a disk-shaped tip in the SECM configuration was simulated by solving an axisymmetric (2D) diffusion problem in cylindrical coordinates (Figure S1). We used COMSOL Multiphysics (version 6.2, COMSOL, Inc., Burlington, MA) to solve the 2D SECM diffusion problem in dimensionless form. The finite element simulation results were generated using COMSOL Multiphysics and were previously published.[S-3-S-5] Specifically, eq S6 for the diffusion of protamine was defined using

dimensionless parameters as

$$\frac{\partial C_{\mathrm{i}}}{\partial \tau} = \frac{\partial^2 C_{\mathrm{i}}}{\partial R^2} + \frac{1}{R}\frac{\partial C_{\mathrm{i}}}{\partial R} + \frac{\partial^2 C_{\mathrm{i}}}{\partial Z^2} \tag{S12}$$

with

$$C_{\mathrm{i}} = \frac{c_{\mathrm{i}}}{c_0} \tag{S13}$$

$$\tau = \frac{Dt}{a^2} \tag{S14}$$

$$R = \frac{r}{a} \tag{S15}$$

$$Z = \frac{z}{a} \tag{S16}$$

The left-hand side of eq S12 was set to zero for the simulation of steady-state approach curves (Figure 7B). In addition, the tip–NE distance, d, was expressed in terms of dimensionless parameters as

$$L = \frac{d}{a} \tag{S17}$$

The distance-dependent tip current was calculated in dimensionless form as follows

$$I(L) = \int_0^1 2\pi R \left(\frac{\partial C_{\mathrm{N}}}{\partial Z}\right)_{Z=0} \mathrm{d}R \tag{S18}$$

The normalized tip current, $i_{\mathrm{T}}/i_{\mathrm{T},\infty}$, was set to 1 at $L = 25$, i.e., equal to $I(L)/I(L = 25)$.

Boundary conditions for the permeant at the membrane (eqs S10 and S11) were also expressed in dimensionless form as

$$\frac{\partial C_{\mathrm{i}}}{\partial Z} = \kappa\lambda\left[\rho C_{\mathrm{i}}\left(1 - \theta_{\mathrm{P}}\right) - \theta_{\mathrm{P}}\right] \tag{S19}$$

$$\frac{\partial \theta_{\mathrm{P}}}{\partial \tau} = \lambda\left[\rho\left(C_{\mathrm{C}} + C_{\mathrm{N}}\right)\left(1 - \theta_{\mathrm{P}}\right) - 2\theta_{\mathrm{P}}\right] \tag{S20}$$

with

$$\lambda = \frac{k_{\mathrm{off}} a^2}{D} \quad \text{(S21)}$$

$$\kappa = \frac{\Gamma_{\mathrm{S}}}{a c_0} \quad \text{(S22)}$$

$$\rho = \beta c_0 \quad \text{(S23)}$$

$$\theta_{\mathrm{P}} = \frac{\Gamma_{\mathrm{P}}}{\Gamma_{\mathrm{S}}} \quad \text{(S24)}$$

where λ is the normalized dissociation constant, κ is the normalized concentration of interaction sites in the NE, and ρ is the normalized strength of protamine–NE interactions in the homogeneous model.

Transient SECM Simulation of 4 µm Tip. We performed the finite element simulation to demonstrate that a 4 µm-diameter pipet yields a lower difference of ~$0.5 i_{\mathrm{T},\infty}$ in transient tip current (Figure S2) than a 10 µm-diameter pipet (Figure 7A).

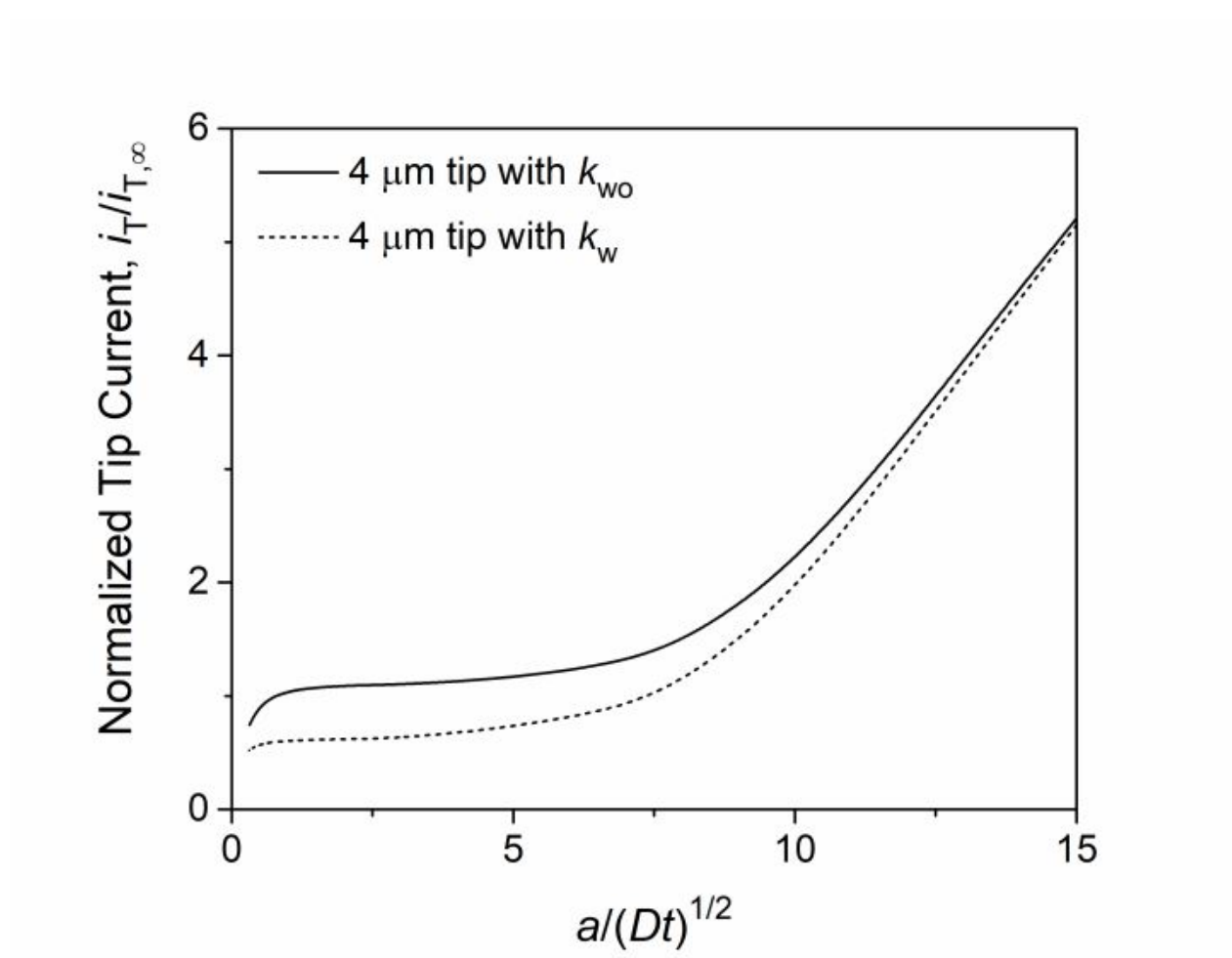

Figure S2. Chronoamperograms as simulated for dissociative protamine transport through NPCs that are free from (solid lines) or saturated with (dotted lines) the nanobody with $k_{\mathrm{wo}} = 6.6$ s^{-1} and $k_{\mathrm{w}} = 2.5$ s^{-1}, respectively.

REFERENCES

(S-1) Solà Colom, M.; Fu, Z.; Gunkel, P.; Güttler, T.; Trakhanov, S.; Srinivasan, V.; Gregor, K.; Pleiner, T.; Görlich, D. A checkpoint function for Nup98 in nuclear pore formation suggested by novel inhibitory nanobodies. *EMBO J.* **2024**, *43*, 2198–2232.

(S-2) Frey, S.; Görlich, D. A new set of highly efficient, tag-cleaving proteases for purifying recombinant proteins. *J. Chromatogr. A* **2014**, *1337*, 95–105.

(S-3) Huang, S.-H.; Amemiya, S. Transient theory for scanning electrochemical microscopy of biological membrane transport: Uncovering membrane–permeant interactions. *Analyst* **2024**, *149*, 3115–3122.

(S-4) Huang, S.-H.; Parandhaman, M.; Jyothi Ravi, M.; Janda, D. C.; Amemiya, S. Nanoscale interactions of arginine-containing dipeptide repeats with nuclear pore complexes as measured by transient scanning electrochemical microscopy. *Chem. Sci.* **2024**, *15*, 15639–15646.

(S-5) Huang, S.-H.; Parandhaman, M.; Jyothi Ravi, M.; Janda, D. C.; Amemiya, S. Nanoscale hydrophobicity of transport barriers in the nuclear pore complex as compared with the liquid/liquid interface by scanning electrochemical microscopy. *Anal. Chem.* **2025**, *97*, 2745–2753.
